# Surgical techniques, open versus minimally invasive gastrectomy after chemotherapy (STOMACH trial): study protocol for a randomized controlled trial

**DOI:** 10.1186/s13063-015-0638-9

**Published:** 2015-03-27

**Authors:** Jennifer Straatman, Nicole van der Wielen, Miguel A Cuesta, Suzanne S Gisbertz, Koen J Hartemink, Alfredo Alonso Poza, Jürgen Weitz, Fransico Mateo Vallejo, Khurshid Ahktar, Ismael Diez del Val, Josep Roig Garcia, Donald L van der Peet

**Affiliations:** Department of Surgery, VU University Medical Center, De Boelelaan 1117, 1081 HV Amsterdam, NL Netherlands; Department of Surgery, Academic Medical Center, Meibergdreef 9, 1105 AZ Amsterdam, NL Netherlands; Department of Surgery, Antoni van Leeuwenhoek Hospital, Plesmanlaan 121, 1066 CX Amsterdam, NL Netherlands; Department of Surgery, Hospital Universitario del Sureste, Ronda del Sur 10, Arganda del Rey, 28500 Madrid, ES Spain; Department of Surgery, Uniklinikum Dresden, Fetscherstraße 74, 01307 Dresden, DE Germany; Department of Surgery, Hospital Jerez de la Frontera, Ronda de Circunvalación, 11407 Cadiz, ES Spain; Department of Surgery, Salford Royal NHS Foundation Trust, Stott lane, Salford, M6 8HD UK; Department of Surgery, Hospital Universitario Basurto, Montevideo Etorbidea 18, 48013 Bilbao, Spain; Department of Surgery, Hospital Universitario de Josep Trueta, Avenida França, 17007 Girona, ES Spain

**Keywords:** Gastric cancer, Gastrectomy, Minimally invasive surgery

## Abstract

**Background:**

Laparoscopic surgery has been shown to provide important advantages in comparison with open procedures in the treatment of several malignant diseases, such as less perioperative blood loss and faster patient recovery. It also maintains similar results with regard to tumor resection margins and oncological long-term survival. In gastric cancer the role of laparoscopic surgery remains unclear.

Current recommended treatment for gastric cancer consists of radical resection of the stomach, with a free margin of 5 to 6 cm from the tumor, combined with a lymphadenectomy. The extent of the lymphadenectomy is considered a marker for radicality of surgery and quality of care. Therefore, it is imperative that a novel surgical technique, such as minimally invasive total gastrectomy, should be non-inferior with regard to radicality of surgery and lymph node yield.

**Methods/Design:**

The Surgical Techniques, Open versus Minimally invasive gastrectomy After CHemotherapy (STOMACH) study is a randomized, clinical multicenter trial. All adult patients with primary carcinoma of the stomach, in which the tumor is considered surgically resectable (T1-3, N0-1, M0) after neo-adjuvant chemotherapy, are eligible for inclusion and randomization. The primary endpoint is quality of oncological resection, measured by radicality of surgery and number of retrieved lymph nodes. The pathologist is blinded towards patient allocation. Secondary outcomes include patient-reported outcomes measures (PROMs) regarding quality of life, postoperative complications and cost-effectiveness. Based on a non-inferiority model for lymph node yield, with an average lymph node yield of 20, a non-inferiority margin of −4 and a 90% power to detect non-inferiority, a total of 168 patients are to be included.

**Discussion:**

The STOMACH trial is a prospective, multicenter, parallel randomized study to define the optimal surgical strategy in patients with proximal or central gastric cancer after neo-adjuvant therapy: the conventional ‘open’ approach or minimally invasive total gastrectomy.

**Trial registration:**

This trial was registered on 28 April 2014 at Clinicaltrials.gov with the identifier NCT02130726.

## Background

Laparoscopic surgery has been shown to provide important advantages in comparison with an open approach in the treatment of gastrointestinal malignant diseases, such as less perioperative blood loss, faster patient recovery and shorter hospital stay. It also maintains similar outcomes with regard to tumor resection margins and oncological long-term survival [1,2]. In gastric cancer, the role of laparoscopic surgery remains unclear.

The current recommended treatment for gastric cancer consists of radical resection of the stomach, with a free margin of 5 to 6 cm from the tumor, combined with a lymphadenectomy. The extent of the lymphadenectomy, performed according to the guidelines of the Japanese Gastric Cancer Association, is considered a marker for radicality of surgery and quality of care [3]. Therefore, it is imperative that a novel surgical approach such as laparoscopic total gastrectomy should be non-inferior with regard to radicality of surgery and lymph node yield.

Several studies have focused on laparoscopic versus open gastrectomy. These studies are predominantly conducted in Asian countries [4,5], where the incidence of gastric cancer is higher in comparison to Western countries [6,7]. The screening program in Japan, which started in 1983, has enabled important advances in the detection and treatment of early gastric carcinomas in this country [8]. As such, tumor stages are lower at the time of diagnosis compared to Western countries, and it is difficult to translate the results of Asian studies to a population for which no screening program exists, and in which the stages of the tumors at diagnosis are higher [9].

Only a few Western studies, one randomized controlled trial and some cohort analyses, have been conducted comparing laparoscopic and open approaches for gastric cancer [10-14]. In the randomized controlled trial by Huscher *et al*., they found that laparoscopic partial gastrectomy showed similar results to open gastrectomy with regards to quality of oncological resection, as measured by the number of retrieved lymph nodes, and five-year survival rate, whereas patient recovery was faster and admission duration was shorter [10-14]. However, these studies are small and underpowered and are exceeded by changes in neo-adjuvant therapies. Further research is indicated in order to establish the optimal surgical strategy.

Moreover, implementation of neo-adjuvant chemotherapy is currently used after the outcome of different studies on this subject [15,16]. Nowadays the use of neo-adjuvant treatment followed by gastric resection is extensive and applies in stage Ib to IVa gastric cancer [17]. The effect of neo-adjuvant chemotherapy on a laparoscopic gastrectomy in comparison with an open resection remains unclear. For instance, in rectal and breast cancer, neo-adjuvant chemotherapy has been associated with response of the tumor and a lower number of lymph nodes found in the specimen [18]. In gastric cancer, preoperative chemotherapy has been associated with a lower number of tumor-positive lymph nodes, however no difference in total lymph node yield was reported [19]. In other series, laparoscopic gastrectomy has shown non-inferior results with regard to lymph node yield in comparison to open gastrectomy, but these studies were conducted before the implementation of neo-adjuvant chemotherapy [12,20,21]. Moreover, the difficulty of dissection and resection, and the quality of a laparoscopically performed esophagojejunostomy, remains a technical challenge. Considering all these factors, such as the differences in populations, the number of retrieved lymph nodes, the location of lymph nodes in anatomical stations, the increased use of neo-adjuvant chemotherapy and the technical difficulties derived from the laparoscopic total gastrectomy, a randomized controlled trial comparing open and laparoscopic total gastrectomy after neo-adjuvant therapy is warranted. Such a trial could provide an answer to the question, ‘Is a minimally invasive total gastrectomy justified in the era of neo-adjuvant chemotherapy?’.

## Methods/Design

### Study objectives

The objective of this study is to establish the optimal surgical strategy in the treatment of patients with gastric cancer. The STOMACH trial is a prospective, international, multicenter, parallel randomized clinical trial. Patients with gastric cancer selected to undergo a total gastrectomy, who have received neo-adjuvant chemotherapy, are randomized between a conventional ‘open’ and a minimally invasive group.

### Endpoints

The primary endpoint is quality of oncological resection with regard to radicality of surgery and lymph node dissection in all the appointed stations. Both the total number of resected lymph nodes and the resected lymph node stations will be examined. After surgery, the surgeon will attach tags with numbers corresponding with the dissected lymph node stations to the specimen. This will allow for a more extensive assessment of the feasibility of minimally invasive versus open resection.

Secondary endpoints include postoperative complications, which are monitored for 30 days postoperatively. Overall length of hospital stay and Intensive Care Unit (ICU) stay will also be recorded. Survival will be monitored for up to three years postoperatively. Quality of life is assessed with patient-reported outcome measures (PROM), the Euro-Quality of Life-5D (EQ-5D) questionnaire, the European Organizaion for Research and Treatment of Cancer Quality of Life Questionnaire 30 (EORTC-QLQ30 and the Stomach 22 module (STO22). Assessment of quality of life will be performed preoperatively, five days postoperatively, three months, six months and one year postoperatively. Cost-effectiveness will be assessed from a hospital and societal perspective.

### Power of the study

The number of dissected lymph nodes in gastric cancer surgery is an important marker for radicality of surgery and quality of care [22-24]. Therefore the primary outcome in this study is the number of retrieved lymph nodes in laparoscopic surgery compared to an open procedure.

It is anticipated that laparoscopic gastric resection will show similar surgical resection specimen quality [19], based on the results of the Dutch Cancer Registry (NKR). The sample size calculation is set to achieve 90% power to detect non-inferiority using a one-sided, two-sample t-test. With a margin of non-inferiority at −4.0 and a significance level (α) of 0.05, the sample size requires 66 patients to be included per group, with a total sample size of 132 patients. A non-inferiority margin of −4.0 is deemed feasible, since the current average lymph node yield at the VU University Medical Center ((VUmc) Amsterdam) is around 20, meaning a lymph node yield of 16 is acceptable.

Since lymph node yield is of interest in cases of radical resection, further correction is necessary for radicality of surgery. The NKR showed that a radical resection was achieved in 79% of patients, although palliative resection figures are not given separately. After correction for radicality, a total of 168 patients are to be included. In other, similar prospective studies, no loss to follow-up was recorded, therefore we do not take into account a percentage for loss to follow-up [25,26].

### Inclusion criteria

All adult patients with primary carcinoma of the stomach, where the tumor is considered surgically resectable (T1-3, N0-1, M0) after neo-adjuvant chemotherapy, are eligible. Only patients with an indication for total gastrectomy are included, in order to exclude bias due to different surgical approaches. Written informed consent is obligatory.

### Exclusion criteria

Exclusion criteria are previous surgery of the stomach and patients with a previous history of cancer or presenting with a co-existing cancer. To allow for appropriate inclusion and randomization, patients operated in an acute setting are excluded. Patients with an American Society of Anesthesiologists (ASA) classification of four of higher are excluded.

### Participating surgeons and clinics

Complication rate, duration of operation and morbidity can be a result of the learning curve of the operating surgeon, and this might bias results. In order to prevent surgeon bias, participating surgeons are to have sufficient experience in open and minimally invasive total gastrectomy. Based on the literature and the Dutch guidelines for gastric carcinoma [27,28], it is required that the participating surgeon has performed at least 20 open and minimally invasive total gastrectomies.

All surgeons in participating centers have sufficient prior experience with both open and minimally invasive gastrectomy. Eight European academic and non-academic centers will participate in the study: the VU University Medical Center, Amsterdam; Academisch Medisch Centrum, Amsterdam; Antoni van Leeuwenhoek Ziekenhuis, Amsterdam; Universitätsklinikum Carl Gustav Carus, Dresden; Hospital Universitario del Sureste, Madrid; Hospital General de Jerez de la Frontera, Cadiz; Salford Royal NHS Foundation Trust, Manchester and Hospital Universitario Basurto, Bilbao.

### Randomization and blinding

Information regarding the study will be provided to the patient at the outpatient clinic. When informed consent is obtained, the patient will be randomized at the outpatient clinic. Randomization occurs via an online module. The participating surgeon can login via a secured module on the STOMACH trial website. Upon filling out the randomization form, an immediate response is obtained, containing a code number and the allocated type of operation.

The study design is unblinded with regard to patient and physician. The patient will be informed about the type of procedure they are allocated to. When patients do not agree to participate in the study they will receive the standard treatment in the corresponding department. The pathologist assessing the specimen is blinded for the operating technique, since radicality of surgery and the number of assessed lymph nodes and lymph node stations is the primary outcome in this trial.

### Data collection and statistics

Data is collected via a secured Internet module and via datasheets on paper. A secured online module has been especially designed for the STOMACH trial, using OpenClinica, version 3.3. © 2015 OpenClinica, LLC. Paper datasheets, such as completed questionnaires, will be sent to the VUmc by mail, where they are kept in a secured room. Data are collected daily until discharge. PROMs are collected preoperatively, five days postoperatively, at three months, six months and one year postoperatively.

One research fellow in the VUmc will monitor the data of all included patients, and maintain regular contact with all participating centers. All required parameters will be collected in an SPSS data file, SPSS version 22, IBM statistics®, Chicago, Illinois, USA . Data analysis will be performed according to the intention-to-treat principle. Continuous variable will be compared with a T-test or Mann-Whitney U as appropriate, and frequencies will be compared with a chi-square or the McNemar analysis as appropriate.

### Ethics

The study is conducted in accordance with the principles of the Declaration of Helsinki and ‘good clinical practice’ guidelines. The independent medical ethics committee of the VUmc (Medisch Ethische Toetsingscommissie VU Medisch Centrum, Amsterdam, the Netherlands) has approved the final version of the protocol prior to the start of the study (approval number: 2014.354 - NL51293.029.14). Written informed consent will be obtained from all participating patients. This trial was registered on 28 April 2014 at Clinicaltrials.gov with the trial number NCT02130726.

### Surgical technique

#### Preoperative preparation

All patients will receive the same preoperative preparation, regardless of allocated treatment. All participating patients will receive standard preoperative prophylactic antibiotics consisting of a single dose of cefuroxime at 1,500 mg and a single dose of metronidazole at 500 mg. Antithrombotic prophylaxis will be administered according to local protocol.

#### Open gastrectomy

For the open gastrectomy, the patient is placed in the supine position. Access to the abdomen is obtained via a median laparotomy. The Omnitract® system, Omni-tract Surgical, St Pauls, Minnesota, USA, is placed over the incision in order to secure vision over the stomach.

#### Minimally invasive gastrectomy

**Figure 1:**
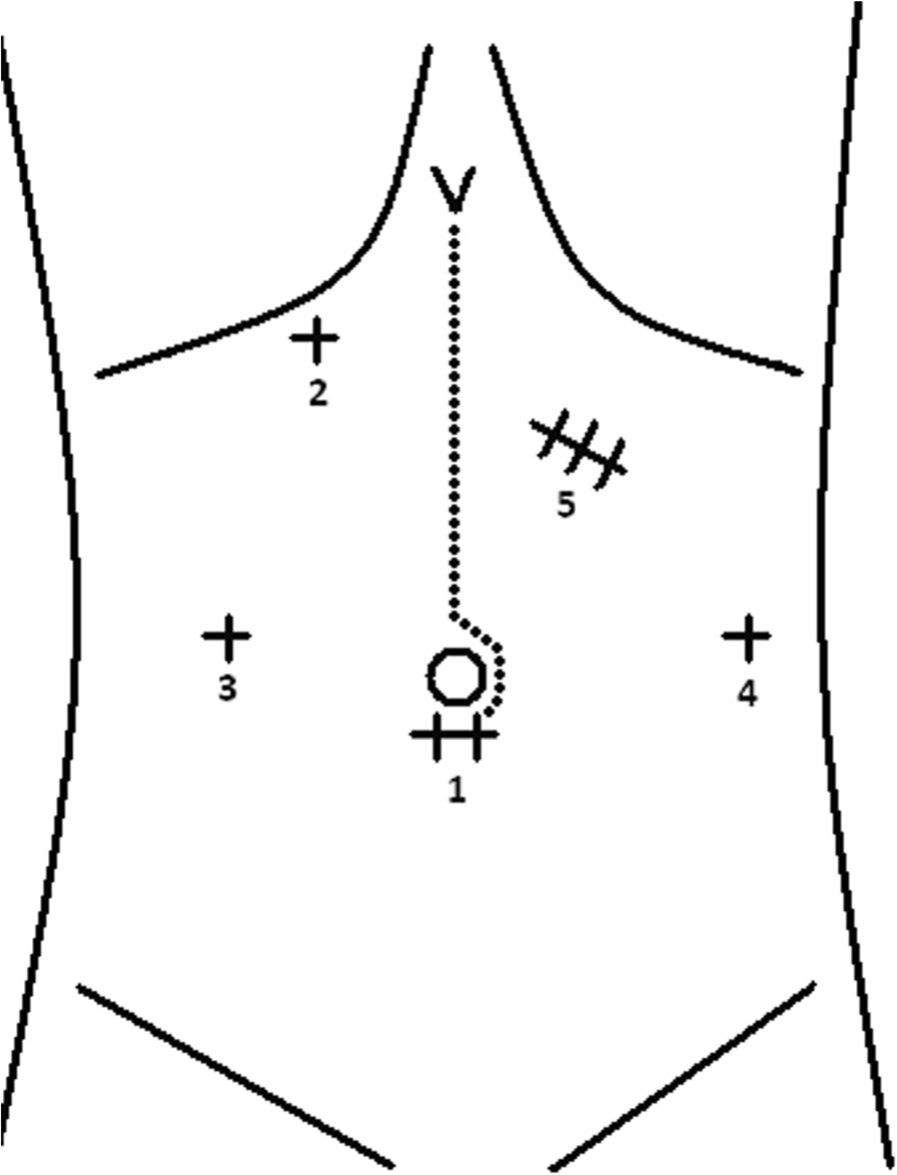
**Overview of placement of trocars in minimally invasive gastrectomy.** 1) laparoscope placement, 2) Nathanson Hook Liver Retractor, (3 and 4) additional instruments, 5) the incision (±5 to 6 cm) is performed in order to allow for retrieval of the specimen, and the wound is protected with an Alexis® wound protector, Applied Medical Resources Corp, Rancho Santa Margarita, California, USA. Dotted line portrays open gastrectomy.

For the minimally invasive gastrectomy, the patient is placed in the reverse Trendelenburg position and the legs are abducted. The surgeon is positioned between the legs of the patient. The first trocar, for the laparoscope, is inserted at the umbilicus. After insertion, a pneumoperitoneum is created. The following trocars are placed with the aid of the laparoscope. The overview of trocar placement is depicted in Figure 1. A Nathanson Hook Liver Retractor® may be placed in order to retract the liver from the operation area.

#### Gastrectomy

After the placement of trocars or opening of the abdomen, the abdomen is inspected for signs of tumor progression. The greater omentum is mobilized and dissected from the transverse colon. Access to the lesser sac is obtained. The right gastro-epiploic artery is identified and clipped and according lymph node stations dissected. This is followed by further dissection and ligation of the right gastric artery and harvesting of the hepatic lymph node stations. The duodenum is dissected up to 5 cm distal to the pylorus, followed by transection of the duodenum.

Dissection continues with mobilization of the left part of the stomach. After identification of the left gastric artery, the artery is clipped and according lymph node stations are harvested. Further dissection continues towards the hiatus, where the pericardial lymph nodes are harvested. After the gastro-esophageal junction is identified and dissected, it is transected using a linear stapling device. With regard to transection, a proximal margin of 6 cm from the tumor is recommended [3]. After *en bloc* resection, the specimen is removed, but not yet stored. After completion of the surgery, the surgeon attaches tags with numbers corresponding to each lymph node station, allowing for separate analysis of each lymph node station.

Reconstruction occurs with a Roux and Y anastomosis. First, the jejunum is mobilized upwards in a retrocolic fashion. Anastomosis between the esophagus and jejunum is performed. Next, a jejunojejunostomy is fashioned. A final overview is performed of the abdomen, with control of hemostasis. Lastly, a silicone drain is placed in the operated area, if deemed necessary by the operating surgeon, and the abdomen is closed.

#### Postoperative management

Irrespective of open or minimally invasive gastrectomy, patients will receive similar postoperative management. Depending on local protocol, a nasogastric tube may be positioned. Oral diet is initiated. Postoperative pain control consists of patient-controlled analgesia (PCA), which is monitored daily by an anesthesiologist. PCA pumps will remain *in situ* for a maximum of three days. Patients are encouraged to be out of bed and walking around the ward, under the guidance of a physiotherapist or nurse. Patients will be discharged when they pass stool, are able to drink, can walk and are comfortable with oral analgesia. A delay in discharge due to ‘social’ reasons will be recorded. Follow-up occurs at the outpatient clinic; patients are seen routinely at three, six and 12 months postoperatively.

## Discussion

Laparoscopic surgery has been shown to provide important advantages in comparison with open procedures in surgery of the rectum and colon. Since the first minimally invasive total gastrectomy in 1996 by Azagra *et al*. [29], several comparative studies between open and minimally invasive approaches of the stomach have been published. Short-term results show less perioperative blood loss, faster patient recovery and earlier discharge from the hospital. One study reported long-term results with similar survival and disease-free survival rates in the open and minimally invasive approach [10].

Most studies are conducted in Asian countries, where a screening program has enabled early detection and treatment. The results of these studies cannot be translated to the Western population. Western studies have deemed minimally invasive gastrectomy to be feasible, although the numbers are small and the studies often underpowered. Furthermore, these studies were conducted before the implementation of neo-adjuvant therapy. Currently, in the Netherlands, less than 10% of patients are operated on via a minimally invasive approach [30]. A prospective, randomized clinical trial is considered necessary in order to establish the optimal surgical technique in gastric cancer: open or minimally invasive gastrectomy.

## Trial status

The Scientific Research Committee of the Cancer Centre Amsterdam, NL, approved the design of the STOMACH trial. The Medical Ethical committee of the VUmc has approved the protocol (approval number: 2014.354 - NL51293.029). The trial is open for recruitment since January 2015.

## Abbreviations

ASA: American Society of Anesthesiologist classification
EORTC: European Organisation for Research and Treatment of Cancer questionnaires
EQ-5D: Euro-Qol (Quality of Life)-5D questionnaire
ICU: Intensive care unit
NKR: Nederlandse Kanker Registratie (Dutch Cancer Registry)
PCA: Patient-controlled analgesia
PROMs: Patient-reported outcome measurements
STOMACH: The surgical techniques, open versus minimally invasive gastrectomy after chemotherapy trial
VUmc: VU University Medical Center
